# Corneal collagen cross-linking (CXL) in thin corneas

**DOI:** 10.1186/s40662-015-0025-3

**Published:** 2015-09-07

**Authors:** Xiangjun Chen, Aleksandar Stojanovic, Jon Roger Eidet, Tor Paaske Utheim

**Affiliations:** SynsLaser Kirurgi, Oslo and Tromsø, Norway; Faculty of Medicine, University of Oslo, Oslo, Norway; Eye Department, University Hospital North Norway, Tromsø, Norway; Department of Medical Biochemistry, Oslo University Hospital, Oslo, Norway; Department of Oral Biology, Faculty of Dentistry, University of Oslo, Oslo, Norway

**Keywords:** Collagen cross-linking, Keratoconus, Thin cornea

## Abstract

Corneal collagen cross-linking (CXL) is a therapeutic procedure aiming at increasing the corneal stiffness in the keratoconus eyes by induction of cross-links within the extracellular matrix. It is achieved by ultraviolet-A (370 nm) irradiation of the cornea after saturation with the photosensitizer riboflavin. In the conventional CXL protocol, a minimum de-epithelialized corneal thickness of 400 μm is recommended to avoid potential irradiation damage to the corneal endothelium. In advanced keratoconus, however, stromal thickness is often lower than 400 μm, which limits the application of CXL in that category. Efforts have been undertaken to modify the conventional CXL procedure to be applicable in thin corneas. The current review discusses different techniques employed to achieve this end and their results. The overall safety and efficacy of the modified CXL protocols are good, as most of them managed to halt the progression of keratectasia without postoperative complications. However, the evidence of safety and efficacy in the use of modified CXL protocols is still limited to few studies with few patients involved. Controlled studies with long-term follow-up are required to confirm the safety and efficacy of the modified protocols.

## Background

Keratoconus is a degenerative disorder of the cornea, characterized by progressive stromal thinning and conical ectasia that result in irregular astigmatism and associated vision loss [1, 2]. It was estimated that the stiffness of a keratoconic cornea is only 60 % of that of the normal cornea, and that the development of conical shape in keratoconus is the result of decreased biomechanical stability [3]. The pathogenesis of keratoconus on molecular level is still uncertain, although it mainly seems to be caused by a reduced number of collagen cross-links and higher pepsin digestion than in normal corneas [3–5]. Reduced mean diameter and interfibrillar spacing of the collagen fibrils [6], slippage of collagen lamellae [7, 8], as well as a loss of the normal interwoven structure of the lamellae [9], have been reported.

Until the introduction of corneal collagen cross-linking (CXL), therapeutic solutions for keratoconus have been limited to the treatment of the consequences of progressive weakening of the cornea – from rigid gas permeable contact lenses (RGP) to corneal transplantation (deep anterior lamellar or penetrating keratoplasty) ultimately [10]. RGP alleviates the symptoms, but does not address the basic defect within the keratoconic cornea, thus the collagen weakening will be unaffected and still continue to progress [10]. Keratoconus remains among the leading indications for penetrating keratoplasty [11], which is a major ophthalmic surgical procedure involving risk for rejection and other serious complications. The 10-year graft survival after penetrating keratoplasty for keratoconus was reported to be 89 % [12].

Corneal crosslinking with riboflavin/ultraviolet-A (UVA, 370 nm), introduced merely a decade ago, is a minimally invasive procedure for treatment of keratectasia via increasing the mechanical and biomechanical stability of the stromal tissue [13–17]. The aim of CXL is to create new chemical bonds (cross-links) between collagen fibrils and other extracellular matrix proteins in the corneal stroma through localized photo polymerization [18]. Exposure of the riboflavin to UVA-irradiation results in absorption of energy and its excitement into a triplet state that undergoes either an aerobic, type 2 reaction, or an anaerobic, type 1 reaction [19]. According to Kamaev and colleagues, an oxygenated environment causes formation of singlet molecular oxygen, which then acts on tissue to produce additional cross-linked bonds. After a quick consumption of oxygen, which occurs only within several seconds, depending on UV-power, temperature, amongst other factors, it is suggested that the main photochemical kinetics mechanism is the direct interaction between the riboflavin triplets and reactive groups of corneal proteins, which leads to the cross-linking of the proteins mainly through radical reactions [19]. These then induce formation of new covalent bonds between the amino acids among the neighboring collagen molecules [17, 20] and among proteoglycan (PG) core proteins, as well as limited linkages between collagen and PG core proteins [21].

The riboflavin also offers a shielding effect to the deeper ocular structures, such as the corneal endothelium, lens, and retina [22], by absorbing the UVA [13, 22]. The critical limitation of CXL in thin corneas is the lack of sufficient corneal thickness for the UVA-radiation to be absorbed and attenuated before it reaches the endothelium. The cell damage threshold of UVA-irradiation combined with riboflavin is 10 times higher than with UVA-irradiation alone [23]. Wollensak et al. [23] demonstrated that when the combination of UVA and riboflavin is used in corneas thinner than 400 μm, the cytotoxicity threshold of 0.35 mW/cm^2^ for the endothelial cell damage can be reached. In conventional CXL procedure, the treatment parameters (0.1 % riboflavin in dextran 20.0 % solution and 3 mW/cm^2^ of UVA for 30 minutes) are assumed to treat the anterior 300 μm of the corneal stroma [24, 25]. Hence, only the patients with a de-epithelialized corneal thickness of at least 400 μm are subjected to this treatment. The downside of this limitation is that eyes with advanced stages of keratectasia often have corneas thinner than 400 μm. Populations of Asian and African origin with inherently thinner corneas [26, 27] may be especially affected by this limitation. Various modifications have been suggested to circumvent that [28–31]. The current review discusses the variety of CXL treatment protocols in thin corneas, as well as their efficacy and safety published in peer-reviewed literature. The results of different CXL protocols in treatment of keratectasia in thin corneas are listed in Table 1.

**Table 1:** Safety of CXL in thin corneas

Author, year	No. of eyes	Surgical procedures	Follow up (months)	VA and topography changes	Endothelial loss	Other complications
Kymionis, *et al.* 2012 [38]	14	Conventional	12	UDVA and CDVA improved, K_mean_ reduced	Yes	No
Hafezi, *et al.* 2009 [30]	20	Hypoosmolar riboflavin solution	6	K_max_ stable or reduced	–	No
Raiskup and Spoerl 2011 [42]	32	Hypoosmolar riboflavin solution	12	CDVA and K_max_ stable	–	No
Wu, *et al.* 2014 [43]	15	Hypoosmolar riboflavin solution	12	1 eye lost 1 line CDVA, the rest remained stable or improved, K_max_ and K_min_ reduced	No	No
Soeters and Tahzib 2015 [48]	13	Hypoosmolar riboflavin solution	12	CDVA improved, K_max_, K_min_ and K_mean_ remain stable	No	No
Gu, *et al.* 2015 [45]	8	Hypoosmolar riboflavin solution	3	CDVA stable, K_max_ stable	Yes	No
Filippello, *et al.* 2012 [28]	20	Transepithelial CXL	18	UDVA and CDVA improved, keratometry values decreased	No	No
Spadea and Mencucci 2012 [31]	16	Transepithelial CXL	6–12	UDVA and CDVA improved, K_max_ reduced	No	No
Kymionis, *et al.* 2009 [29]	2	Custom epithelial debridement	9	Topography stable	No	No
Kaya, *et al.* 2011 [69]	2	Custom epithelial debridement	1	–	–	No
Mazzotta and Ramovecchi 2014 [70]	10	Custom epithelial debridement	12	UDVA, CDVA stable, K_mean_ reduced	No	No
Jacob, *et al.* 2014 [73]	14	Contact lens-assisted	6–7	CDVA stable, K_max_ stable	No	No

*K*_*max*_ = Maximum keratometric reading, *K*_*min*_ = Minimum keratometric reading, *K*_*mean*_ = Mean keratometry reading, *SimK* = Simulated keratometry, *VA* = Visual acuity, *UDVA* = Uncorrected distance visual acuity, *CDVA* = Corrected distance visual acuity

## Review

### Conventional collagen cross-linking

The conventional CXL procedure as described in the Dresden protocol in 2003 [17], its modified version in 2008 [32], and the Siena protocol [33] applies to corneas with minimal stromal thickness of 400 μm, and involves the removal of the central 7–9 mm of corneal epithelium followed by instillation of isoosmolar riboflavin 0.1 % solution in 20 % dextran. UVA (370 nm) irradiation with 3 mW/cm^2^ of UVA for 30 minutes (5.4 J/cm^2^) over 8 mm diameter of central cornea is initiated after stromal saturation with riboflavin. The efficacy of this protocol is supported by numerous studies since its introduction in 2003 [17, 34–37].

Kymionis et al. [38] applied conventional CXL procedure in 14 thin corneas with minimum corneal thickness of less than 400 μm (range 340–399 μm) after epithelial removal. Improvement in uncorrected distance visual acuity (UDVA), corrected distance visual acuity (CDVA), and reduction in mean keratometry readings were recorded during the 12 months follow-up. However, despite the absence of clinically evident complications, significant reduction of endothelial cell density from 2733 to 2411 cells/mm^2^ was observed postoperatively. The film of 0.1 % isoosmolar riboflavin with 20 % dextran was measured to be approximately 70 μm thick after 1 minute of instillation and remained stable for 22 minutes [39]. With the riboflavin-dextran film, the UVA irradiance in human corneal stroma at 400 μm was measured to be 0.21 mW/cm^2^, which is much lower than the previously mentioned cytotoxicity level on which the set limitation of minimal deepithelialized stromal thickness of 400 μm is based. Hence, the absorption and shielding of UVA by the riboflavin film may have prevented the damage to the endothelium. Nevertheless, longer follow-up and larger patient series is essential to evaluate the safety and efficacy of conventional CXL in clinical application in thin corneas.

### Hypoosmolar riboflavin solution

The cornea has an inert swelling pressure [40], meaning that the corneal stroma has the tendency to increase its volume in an isooncotic environment. The deepithelialized cornea can swell to double its normal thickness when irrigated with a hypoosmolar solution [41]. Hafezi and co-workers [30] applied this method to increase corneal thickness before CXL in thin corneas. After epithelial removal, 0.1–20 % dextran isoosmolar riboflavin was applied to the cornea for 30 minutes. The 0.1 % dextran-free hypoosmolar riboflavin was then administered until the corneal thickness at the thinnest point reached 400 μm, before the initiation of UVA irradiation. The authors reported a stabilization of keratectasia in 20 eyes treated with this approach. A later study by Raiskup et al. [42] applied 0.1 % hypoosmolar riboflavin after epithelial debridement until the riboflavin saturated cornea reached the minimum of 400 μm. In this study, one year after the treatment, CDVA and keratometric value remained unchanged and no damage to the cornea in the form of detectable scarring lesions in the stroma was registered. Similar results were reported by Wu et al. [43] On the contrary, in eyes treated with isoosmolar riboflavin solution, a permanent stromal scar tended to develop in thin corneas after CXL [44]. Gu et al. [45] used 0.1 % hypoosmolar riboflavin solution as saturation and swelling solution in 8 thin corneas that underwent CXL procedure. They reported a slight decrease of endothelial cell density 3 months after the treatment.

The preoperative swelling of the cornea broadens the spectrum of CXL indications to thinner corneas. However, Hafezi and colleagues [46] reported a case where CXL could not stop the progression of keratoconus in a very thin cornea (minimal thickness of 268 μm after removal of the epithelium), despite the fact that swelling with hypoosmolar riboflavin solution increased the thickness to 406 μm and no adverse endothelial reaction was observed postoperatively. The authors, therefore, hypothesized that there is a minimal, yet to be determined stromal thickness necessary for effective CXL to occur. They suggested a minimal stromal thickness of 330 μm or more before swelling, when using hypoosmolar riboflavin solution.

Kaya et al. [47] and Soeters et al. [48] performed intraoperative corneal thickness measurements during CXL with hypoosmolar riboflavin solution in thin corneas. They found that the artificial swelling effect was transient, and the thinnest pachymetric readings decreased significantly after 10 and 30 minutes of isoosmolar riboflavin (with dextran) application, with or without UVA irradiation. Thinning of deepithelialized cornea after instillation of 0.1–20 % dextran riboflavin isoosmolar solution has also been reported in other studies [49, 50]. The authors inferred that the reduction of the corneal thickness was induced by the hyperoncotic effect of the dextran. Vetter et al. [51] evaluated the modulatory effect of various riboflavin 0.1 and 0.2 % compositions on the central corneal thickness in fresh postmortem porcine eyes. No correlation between the osmolarity of the composition and the swelling behavior of the treated corneas was observed, whereas an inverted correlation was verified between the dextran concentration and the swelling effect. Concurrently, lower absorption and shielding effect of the thinner hypoosmolar riboflavin film on the cornea, by application of the hypoosmolar riboflavin without dextran alone, would increase irradiance level in the stroma, putting the endothelium at higher risk [39]. Therefore, the cornea should be swollen to a thickness greater than 400 μm or concentration of riboflavin in the hypoosmolar solution could be increased. It was therefore suggested that development of new riboflavin solutions with isooncotic properties to create a stable film could increase the safety of CXL [50]. Moreover, lack of the evaporation resistance provided by the corneal epithelium [52], and/or an increase in endothelial pump activity may also contribute to corneal thinning [53–55]. It was proposed that removal of the lid speculum during riboflavin saturation, and use of irradiating devices with shorter irradiation time (and higher power) might be advantageous [47, 50, 54, 55]. Monitoring the corneal thickness throughout CXL treatment could also be important. CXL can be expected to have less effect on biomechanics of artificially swollen corneas due to the lower relative concentration of collagen in the hydrated stroma [56, 57]. Long-term follow-up studies addressing this issue are warranted.

### Transepithelial collagen cross-linking

Substances such as benzalkonium chloride, ethylenediaminetetraacetic acid (EDTA) and trometamol, especially when combined, enhance epithelial permeability of hydrophilic macromolecules, such as riboflavin [58–61]. By adding the enhancers to help riboflavin penetrate to the corneal stroma through the intact epithelium, CXL can be performed without epithelial debridement (transepithelial CXL) [28]. Transepithelial CXL has been proposed (but not proven) to reduce early postoperative pain, temporary worsening of vision, as well as complications such as infectious keratitis after conventional CXL [62]. Additionally, thinner corneas may be treated safer by transepithelial compared to the conventional CXL, since the endothelium is better protected by UVA-filtering effect of the intact epithelium.

In a bilateral study, Filippello et al. used trometamol and sodium EDTA as enhancers and applied transepithelial CXL in 20 keratectatic eyes with a mean corneal thickness (including epithelium) of 412 ± 21 μm [28]. The transepithelial CXL treatment appeared to halt the progression of keratoconus in all treated eyes over 18 months follow-up. It also yielded statistically significant improvements in all visual and topographic outcome measures, whereas the contralateral untreated eyes demonstrated worsening of all parameters. Spadea et al. [31], who used a similar protocol in thin corneas, confirmed its effect in stabilization of the keratoconic eyes. However, the visual and topographic improvement was minimal. No endothelial cell damage was observed in either of the studies.

Wollensak et al. estimated a 64 % increase in corneal rigidity in human corneas with transepithelial CXL using topical anesthetics and benzalkonium chloride as enhancers, versus a 320 % increase when using CXL with de-epithelialization [63]. The safety and reproducibility of the study by Filippello et al. have recently been questioned [64] since the postoperative demarcation line depth in their study [28] was only approximately 100 μm, in contrast to about 300 μm in conventional CXL with epithelial debridement. Seiler and Hafezi [24] first reported the demarcation line after CXL and related the depth of the line to that of keratocyte death after CXL as measured by confocal microscopy [65]. They suggested that the line represented the transition zone between cross-linked anterior and untreated posterior stroma. It is unclear whether the shallower demarcation line using the transepithelial approach was due to limited penetration of riboflavin into the stroma or that it was a result of reduced UVA-light penetration by shielding from riboflavin-impregnated intact corneal epithelium. Iontophoresis-assisted transepithelial CXL, using a noninvasive delivery system based on a small electric current, was recently designed to enhance the penetration of riboflavin into the corneal stroma [66]. Preclinical results showed that the iontophoresis was able to increase the concentration of riboflavin in the corneal stroma when compared to enhancer-assisted transepithelial CXL, but did not reach concentrations previously reached with conventional epithelium-off CXL. Demarcation line after iontophoresis-assisted transepithelial CXL appeared to be less easily distinguishable and shallower than in conventional CXL, however, it demonstrated features more similar to that after conventional CXL in terms of depth and visualization, compared to enhancer-assisted transepithelial CXL [63, 67]. In general, there is consensus within the scientific community that current transepithelial CXL protocols are not as effective as conventional epithelium-off CXL [60, 61, 68].

### Custom epithelial debridement technique

Kymionis et al. [29] performed CXL with custom pachymetry-guided epithelial debridement in one keratoconic eye and one post-LASIK keratectatic eye with thinnest stroma of less than 400 μm. In this modified CXL approach, 8.0 mm diameter of corneal epithelium was removed; leaving a small, localized area of corneal epithelium corresponding to the thinnest area over the apex of the cone. The authors suggested use of hypoosmolar riboflavin during the UVA-irradiation to avoid corneal stromal dehydration as well as to maintain the stromal riboflavin concentration. Nine months postoperatively, topography remained stable, and no endothelial cell density alteration was detected in the treated eyes. However, a later study by Kaya et al. [69] suggested that the epithelium over the cone area spared the stroma underneath from the CXL effect. Four weeks after the treatment, stromal haze and demarcation line were detected in the corneal areas with epithelial debridement, but not in the areas with intact epithelium; deepithelialized stroma outside the cone region displayed total keratocyte apoptosis and honeycomb-like edema, whereas it was minimal beneath the intact epithelium [69]. In contrast, Mazzotta *et al.* [70] demonstrated keratocyte apoptosis at an average depth of 160 μm under the epithelial island compared to 250 μm under the de-epithelialized area in 10 eyes with 1-year follow-up.

One previous study demonstrated that the stromal uptake of riboflavin after grid pattern of full-thickness epithelial debridement was heterogeneous, with full penetration to the stroma immediately beneath the areas of epithelial debridement and no penetration to the stroma beneath the intact epithelium [71]. Inadequate riboflavin saturation together with the ability of the epithelium to absorb the UVA radiation [72] may lead to reduced CXL effect in the cone area and affect the efficacy of the whole procedure. Long-term efficacy of this modified CXL procedure with a larger number of patients needs to be assessed.

### Contact lens-assisted collagen cross-linking

Contact lens-assisted CXL (CACXL) was introduced by Jacob et al. [73] A Soflens daily disposable soft contact lens (14 mm diameter, 8.6 mm basal curvature; Bausch & Lomb) of 90 μm thickness made of hilafilcon and without UV filter was immersed in isoosmolar riboflavin 0.1 % in dextran for 30 minutes, before it was applied onto the deepithelialized, riboflavin-saturated cornea. The UVA-radiation of 3.0 mW/cm^2^ for 30 minutes was initiated after the confirmation that the minimum corneal thickness including the contact lens and riboflavin film was greater than 400 μm. The riboflavin solution was instilled every 3 minutes during the UVA-radiation to maintain corneal saturation and to keep the pre-corneal and pre-contact lens riboflavin film uniform. The pre-corneal riboflavin film with contact lens created an absorption medium in the pre-corneal space by artificially increasing the thickness of the “riboflavin-filter”.

In the 14 eyes treated with CACXL, the authors reported an average increase of the minimum corneal thickness by 108 μm if the contact lens and riboflavin film were included. At a mean follow-up time of 6.1 ± 0.3 months (range: 6–7 months), the mean postoperative depth of the stromal demarcation line was measured at 252.9 μm. No significant endothelium loss or signs of postoperative endothelial damage were observed. No significant change in the CDVA, or mean maximum keratometric value was detected postoperatively, although 1 D decrease of maximum keratometric value was observed in 4 eyes (28.5 %).

The advantage of the CACXL is that it is not dependent on the swelling properties of the cornea and that the cornea is not subjected to edema, which may cause Descemet membrane folds and endothelial damage. However, the surface irradiance at the level of the corneal stroma is reduced by 40–50 % in CACXL secondary to absorption by the riboflavin film and soaked contact lens. Furthermore, oxygen diffusion, which has been demonstrated to be crucial in the CXL process, might be hindered by the contact lens. As a result, the effect of CXL may be reduced. The small patient population, short follow-up and absence of a control group are the limitations of the study.

## Conclusion

A minimum corneal thickness of 400 μm is recommended in conventional CXL treatment. With improved screening technique in keratoconus diagnosis, most of the keratoconus eyes would be able to be treated by this protocol. However, late diagnosed progressive keratoconus eyes often have values below this threshold. To offer CXL to this critical group of patients, several modifications have been proposed. The overall safety of the presented protocols for CXL in thin corneas is good, as most of them managed to halt the progression of keratectasia without postoperative complications. Furthermore, modification of the tonicity and concentration of the photosensitizing riboflavin and modification of the UV energy and/or power have been proposed. Iseli et al. [74] suggested that a higher riboflavin concentration may be applied for improved protective screening of the endothelium in thin corneas. Accelerated CXL (UVA irradiation at 30 mW/cm^2^ for 3 minutes) has recently been reported to stabilize the progression of keratoconus in 34 thin corneas, without endothelial cell density loss during the 12 months of follow-up [75]. Furthermore, in accelerated CXL, pulsed UV light seems to result in a higher effect compared to continuous UV light, presumably due to optimization of oxygen availability [76]. Oxygen concentrations measured in the corneal stroma showed that the certain combination of “on” and “off” time would facilitate continuous replenishment of oxygen [77], leading to increased CXL effect without the necessity to increase UV energy [78]. Thus, using the pulsed mode during UVA irradiation may maximize the efficacy of CXL while maintaining or improving the safety profile of the procedure, which may be especially beneficial in treating thin corneas.

Ideally, a comprehensive mathematical model should be introduced to calculate an optimal set of parameters such as concentration and tonicity of Riboflavin, as well as UV-light-power, duration and dose for any given corneal thickness. That way not only the limitation of the treatment in thin corneas will be addressed, but a customized set of parameters could lead to addressing specific needs of any individual patient. At this point, only laboratory research can be found on the subject [79, 80].

The evidence of safety and efficacy regarding the use of modified CXL protocols is still limited to a handful of studies. Future long-term follow-up studies with a larger number of participants are warranted.

## Abbreviations

CDVA: Corrected distance visual acuity
CXL: Corneal collagen cross-linking
CACXL: Contact lens-assisted CXL
EDTA: Ethylenediaminetetraacetic acid
PG: Proteoglycan
UDVA: Uncorrected distance visual acuity
UVA: Ultra-violet A
